# Genome sequence of *Bacillus subtilis* NGII: a rhizobacteria from potato plants

**DOI:** 10.1128/mra.01025-25

**Published:** 2025-12-29

**Authors:** Anastasia Nikolaeva, Daria Pudova, Guzel Lutfullina, Marat Lutfullin, Yaw Akosah, Ayslu Mardanova

**Affiliations:** 1 Department of Microbiology, Institute of Fundamental Medicine and Biology, Kazan Federal University 473102, Kazan, Russia; 2 Laboratory of Agrobioengineering, Institute of Fundamental Medicine and Biology, Federal University 473102, Kazan, Russia; 3 Department of Molecular Pathobiology, College of Dentistry, New York University 5894, , New York, New York, USA; University of Wisconsin-Madison, Madison, Wisconsin, USA

**Keywords:** genome sequence, *Bacillus subtilis*, rhizobacteria

## Abstract

We report the whole-genome sequence of *Bacillus subtilis* strain NGII isolated from the rhizospheric soil. Genomic insights deepen understanding of the strain’s potential as a biostimulant in plant protection. Sequencing and assembly yielded a 4.1 Mb genome for *B. subtilis* NGII with an average GC content of 43.5%.

## ANNOUNCEMENT

Various plant-associated *Bacillus* spp. have been successfully applied as biological stimulants that improve plant protection and growth promotion (1). The biocontrol properties of *Bacillus* spp. are due to a wide range of secondary metabolites, such as polyketides, terpenes, siderophores, and antimicrobial lipopeptides. It has been discovered that the production of nonribosomal peptides varies among different *Bacillus* strains isolated from the same soil samples (2, 3).

Here, we present the whole-genome sequence of the novel *B. subtilis* strain NGII isolated from the rhizosphere of potato plants located in Kazan, Russia (coordinates N55.615850°, E49.335570°). Briefly, the root material was collected and separated from excess soil by shaking. The roots (5 g) were placed in 50 milliliters of sterile phosphate-buffered saline solution and vigorously stirred to remove any rhizospheric soil attached to the root surfaces. A series of dilutions of the soil suspension were prepared and plated on Luria-Bertani agar (10.0 g tryptone, 5.0 g yeast extract, 5.0 g NaCl, 20 g agar, and 1,000 mL water) and incubated for 24 h at 30°C (4). Pure bacterial culture was grown in Luria-Bertani broth at 30°C overnight (180 rpm). Genomic DNA was extracted using the GeneJET Genomic DNA Purification Kit (Thermo Scientific, #K0722) following the default manufacturer’s protocol, and WGS libraries were prepared with the Illumina DNA Prep (M) Tagmentation default protocol. Sequencing was performed on the NovaSeq 6000 system (Illumina) to generate 2 × 150 bp paired-end reads. As a result, 16,026,818 reads were used for genome assembly. After evaluating the sequencing quality with FastQC v0.12.1, raw reads were filtered using Trimmomatic v0.39 (parameters: HEADCROP:14, SLIDINGWINDOW:4:20, MINLEN:30). High-quality reads were assembled using SPAdes v. 4.0.0 with “--isolate” parameter (5). Statistics of genome assembly was performed with QUAST v. 5.2.0 and represented as 22 scaffolds, with a total length of 4.1 Mb (G + C content, 43.5%; genome coverage, 328×; N50, 1.1 Mb; and L50, 2 bp). Then, the assembled genome was annotated using RAST with default parameters and revealed 4,201 genes (total) with 4,054 coding genes, 45 tRNAs, 5 rRNAs, 5 ncRNAs, and 92 total pseudogenes.

For strain identification, the whole genome sequence of *B. subtilis* NGII was analyzed using the JSpeciesWS web server (6) to calculate average nucleotide identity (ANI). The analysis, which included comparisons with 21 complete genomes from eight *Bacillus* species, confirmed that *B. subtilis* NGII belongs to the species *B. subtilis*, showing ANI values >98.5%. AntiSMASH_8.0.2 analysis (7) identified gene clusters associated with the synthesis of secondary metabolites such as plipastatin, bacilysin, subtilosin A, bacillibactin, fengycin, bacillaene, and surfactin in the genome of *B. subtilis* NGII (Table 1). BAGEL4 (8) revealed sactipeptides, sporulation-killing factor (*skfA*), and subtilosin (*sboX*) in the genome. The genome sequence was submitted to the National Center for Biotechnology Information (NCBI) and annotated by the NCBI Prokaryotic Genome Annotation Pipeline v6.10. Default parameters were used except where otherwise noted.

**TABLE 1:** List of putative secondary metabolites in *Bacillus subtilis* strain NGII

Contig	Region	Type	From	To	Most similar known cluster	Biological effects
1	1.1	Compound likely used as a terpene precursor (terpene-precursor)	639,366	660,256	–^*a*^	–
1	1.2	Type III PKS	841,671	882,768	1-carbapen-2-em-3- carboxylic_acid_biosynthetic_gene	–
1	1.3	Terpene	942,229	964,127	–	–
1	1.4	Non-ribosomal peptide synthetase	1,038,467	1,087,158	Plipastatin	Antifungal (9)
2	2.1	RiPP precursor recognition element (RRE-element) containing cluster	124,050	144,319	–	–
2	2.2	Cluster containing a secondary metabolite-related protein that does not fit into any other category	351,881	393,299	Bacilysin_biosynthetic_gene_cluster	Antibacterial, antifungal (10, 11)
2	2.3	Sactipeptide	396,085	417,696	Subtilosin_A_biosynthetic_gene_cluster	Antibacterial, antifungal (10, 12)
2	2.4	Non-ribosomal peptide metallophores, non-ribosomal peptide synthetase, compound likely used as a terpene precursor (terpene-precursor)	932,463	998,865	Bacillibactin_biosynthetic_gene_cluster	Iron acquisition (9, 10)
3	3.1	Non-ribosomal peptide synthetase, beta-lactone containing protease inhibitor (betalactone)	1	27,569	Fengycin_biosynthetic_gene_cluster	Antifungal, antibacterial (10, 13)
3	3.2	Trans-acyltransferase polyketide synthase (trans-AT PKS), non-ribosomal peptide synthetase, Type III PKS, other types of PKS	100,454	215,190	Bacillaene_biosynthetic_gene_cluster	Bacteriostatical (9, 10)
3	3.3	Terpene	807,093	827,896	–	–
4	4.1	Sactipeptide, ranthipeptide	26,240	49,193	Sporulation_killing_factor _biosynthetic_gene_cluster	Lysis of sibling cells (14)
4	4.2	Non-ribosomal peptide synthetase	183,196	248,587	Surfactin_biosynthetic_gene_cluster	Antibacterial, antiviral, hemolytic, swarming motility, and biofilm formation (9, 10, 13)

^*a*^ “_” was used to depict the absence of a cluster.

In conclusion, the resulting genome analysis of the rhizospheric strain *B. subtilis* NGII provides valuable insights into its potential application in agriculture for enhancing plant protection and growth promotion.

## Data Availability

Data of whole genome shotgun sequencing project were uploaded to the NCBI database under accession number JBPJSK000000000, BioProject accession number PRJNA1282471, and BioSample accession number SAMN49650439. Accession number for the raw reads SRR35238732.
